# Chronic lipopolysaccharide exposure induces cognitive dysfunction without affecting BDNF expression in the rat hippocampus

**DOI:** 10.3892/etm.2014.1479

**Published:** 2014-01-08

**Authors:** BIN ZHU, ZHI-GANG WANG, JIE DING, NING LIU, DA-MING WANG, LIANG-CAI DING, CHUN YANG

**Affiliations:** 1 Department of Critical Care Medicine, The Third Affiliated Hospital of Soochow University, Changzhou, Jiangsu 213003, P.R. China; 2 Department of Respiratory Medicine, The Third Affiliated Hospital of Soochow University, Changzhou, Jiangsu 213003, P.R. China; 3 Department of Anesthesiology, The Third Affiliated Hospital of Soochow University, Changzhou, Jiangsu 213003, P.R. China

**Keywords:** lipopolysaccharide, cognitive dysfunction, pro-inflammatory cytokines, amyloid-β, brain-derived neurotrophic factor

## Abstract

Previous studies have shown that lipopolysaccharide (LPS) has the potential to cause cognitive dysfunction. However, the underlying pathogenesis has yet to be fully elucidated. Increasing attention is being focused on infection in the central nervous system. Therefore, the present study aimed to investigate the behavioral performance of rats receiving intraperitoneal injections of LPS and to determine the expression levels of amyloid-β (Aβ), brain-derived neurotrophic factor (BDNF) and pro-inflammatory cytokines in the hippocampus. In total, 30 male Wistar rats were randomly divided into 3 groups (each n=10): Control and 3 and 7 day LPS administration groups. The rats were intraperitoneally injected with saline or LPS for 3 or 7 days. Following this, rats performed the Morris water maze test, in which the latency to the platform and proportion of time spent in the target quadrant were recorded. Rats were then sacrificed and the hippocampi were harvested for determination of interleukin (IL)-1β, IL-6, tumor necrosis factor-α (TNF-α), Aβ and BDNF expression levels. LPS administration for 3 and 7 days significantly increased the latency to the platform and decreased the proportion of time spent in the target quadrant compared with those in the control group, (P<0.05). Administration of LPS for 3 and 7 days induced statistically significant increases in the expression levels of IL-1β, IL-6 and TNF-α in the hippocampus, compared with those in the control group (P<0.05). Additionally, the administration of LPS for 7 days induced a statistically significant increase in the expression level of Aβ in the hippocampus, compared with that in the control group (P<0.05). However, the administration of LPS did not elicit a statistically significant change in the expression level of BDNF in the hippocampus, compared with that in the control group (P>0.05). The results indicate that LPS induces cognitive dysfunction, which is associated with increased expression levels of pro-inflammatory cytokines and Aβ, but does not affect the expression of BDNF in the hippocampus.

## Introduction

Cognitive dysfunction is a symptom characterized by dysfunction in intellectual performance and learning (1,2). However, its pathogenesis has yet to be fully elucidated. Increasing evidence has shown that infection of the central nervous system is associated with the pathogenesis of cognitive dysfunction.

Lipopolysaccharide (LPS) is a cell wall component of Gram-negative bacteria and induces neuronal death, inhibits neurogenesis and impairs synaptic plasticity and memory (3–6). Previous studies have indicated that peripheral administration of LPS causes functional impairments in the brain (7,8). LPS-induced peripheral infection activates the immune system, which conveys a message to the brain causing the production of inflammatory cytokines. Excessive expression of pro-inflammatory cytokines in the brain may cause behavioral deficits (9,10). Regulating the inflammatory response in the brain following a peripheral infection may be important in protection against behavioral disorders (7). Moreover, an LPS-induced inflammatory response is characterized by an increased expression of pro-inflammatory cytokines, which include interleukin (IL)-1β and tumor necrosis factor-α (TNF-α). Chronic activation of pro-inflammatory cytokines has been indicated to be a pivotal factor in the development of cognitive impairment (11–14). Although large studies have raised the possibility that pro-inflammatory cytokines are implicated in cognitive impairments induced by the peripheral administration of LPS, other studies have not found that cognitive deficits are improved following antibiotic treatment (15,16).

In addition, a possible mechanism by which the peripheral inflammatory response may affect cognitive function is via interference with the expression of amyloid-β (Aβ) and brain-derived neurotrophic factor (BDNF) (17,18). The aim of the present study was to investigate the behavioral performance of rats receiving intraperitoneal injections of LPS and to determine the expression levels of Aβ, BDNF and pro-inflammatory cytokines in the hippocampus.

## Materials and methods

### Animals and drugs

In total, 30 male Wistar rats weighing 180–220 g were purchased from the Shanghai Animal Center (Shanghai, China). The rats were housed five per cage with access to food and water *ad libitum* and were maintained on a 12-h light/dark cycle (lights on at 07:00 a.m.). Rats were randomly divided into three groups (n=10 each) and were intraperitoneally administered saline or LPS (Sigma-Aldrich, St. Louis, MO, USA) at a dose of 250 μg/kg for 3 or 7 days consecutively. The experimental procedures were approved by the Institutional Animal Ethics Committee of Soochow University (Changzhou, China).

### Morris water maze

Following intraperitoneal injections of LPS for 3 or 7 days, the Morris maze test was conducted to measure the cognitive function of the rats. As previously described (19), the water maze model was performed in a circular tank (diameter, 1 m) filled with water. A platform was submerged below the surface of the water in the center of the target quadrant. The swimming paths of the rats were recorded by a video camera and analyzed by Videomot software (Huaibei Zhenghua Biologic Apparatus Facilities Co., Ltd., Huaibei, China). Rats were placed in the maze from four random points of the tank and were allowed to search for the platform for 60 sec. However, if this was not achieved, the rat was gently placed on the platform and left for 10 sec. The latency to the platform and the proportion of time spent in the target quadrant were recorded.

### Determination of IL-1β, IL-6 and TNF-α expression levels

Following the behavioral test, rats were immediately sacrificed by decapitation and the hippocampi were harvested. BDNF, IL-1β, IL-6 and TNF-α expression levels in the hippocampus were measured using a sandwich-ELISA with anti-BDNF, IL-1β, IL-6 and TNF-α antibodies, according to the manufacturer’s instructions (Nanjing Jiancheng Bioengineering Institute, Nanjing, China). The hippocampi were homogenized in phosphate buffer solution with 1 mM phenylmethylsulfonyl fluoride and 1 mM ethylene glycol-O,O’-bis(2-aminoethyl)-N,N,N′,N′-tetraacetic acid. Microtiter plates (96-well; flat-bottom) were coated for 24 h with the samples and diluted 1:2 in sample diluent. The standard curve ranged between 7.8 and 500 pg/ml. Plates were washed three times with sample diluent and then monoclonal rabbit antibodies, that were diluted 1:200 in sample diluent, were added to each well. The plate was then incubated for 2 h at room temperature. After washing, peroxidase-conjugated anti-rabbit antibodies (1:2,000) were added to each well and the plate was incubated at room temperature for 1 h. Following the addition of streptavidin-enzyme, substrate and stop solution, the levels of BDNF, IL-1β, IL-6 and TNF-α were determined by absorbance at 450 nm. The standard curve demonstrated a direct relationship between optical density and BDNF, IL-1β, IL-6 and TNF-α concentration. Total protein was measured by the Lowry method, using bovine serum albumin as a standard.

### Determination of Aβ expression levels

Total RNA was isolated from frozen muscle biopsy tissues using TRIzol reagent (Tiangen Biotech Co., Ltd., Beijing, China), according to the manufacturer’s instructions. The concentration of total RNA was measured by spectrophotometry and reverse-transcribed with an RT-PCR kit (Tiangen Biotech Co., Ltd.). Quantitative PCR was performed using a SYBR Green I kit (Tiangen Biotech Co., Ltd.). Primer sequences were as follows: Aβ forward, 5′-CCAGCCAATACCGAAAATGA-3′ and reverse, 5′-TGATGTTTGTCAGCCCAGAA-3′; and β-actin forward, 5′-CCTGTGCTGCTCACCGAGGC-3′ and reverse, 5′-GACCCCGTCTCTCCGGAGTCCATC-3′. PCR conditions were 50°C for 2 min, 95°C for 10 min and 40 cycles at 95°C for 15 sec and 60°C for 60 sec.

### Statistical analysis

Data are expressed as mean ± SD. Statistical analyses were performed by one-way analysis of variance and post hoc analyses were performed using Fisher’s least significant difference tests. Statistical analyses were conducted using Statistical Product for Social Sciences (SPSS), version 17.0 (SPSS, Inc., Chicago, IL, USA). P<0.05 was considered to indicate a statistically significant difference.

## Results

### Behavioral performance in the Morris water maze

**Figure 1:**
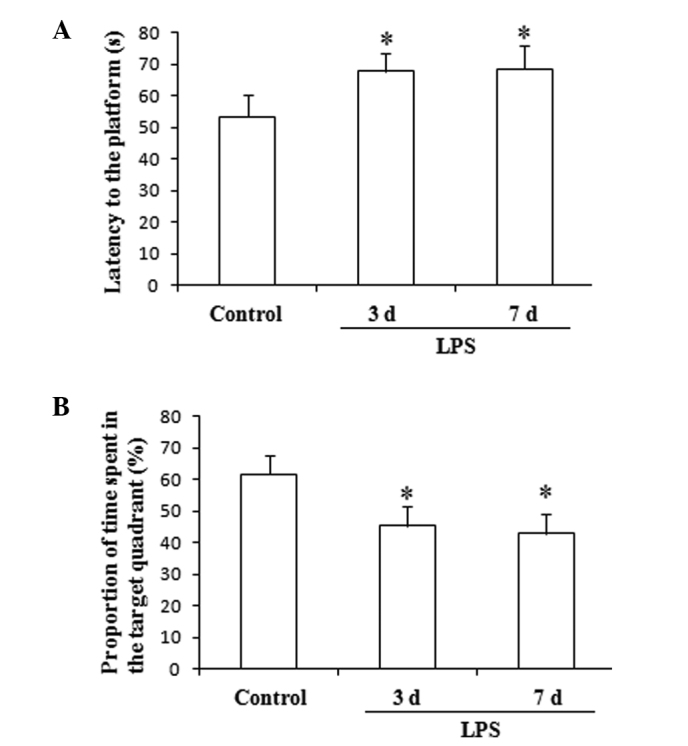
Behavioral performance of rats in the Morris water maze. Effect of LPS on the (A) latency to the platform and (B) proportion of time spent in the target quadrant. ^*^P<0.05, vs. control. LPS, lipopolysaccharide.

The results of this test, which are presented in Fig. 1, indicate that the administration of LPS for 3 and 7 days significantly increased the latency to the platform and decreased the proportion of time spent in the target quadrant, compared with the control group (F_(2,27)_, 11.75; P<0.05; Fig. 1).

### Expression levels of pro-inflammatory cytokines in the hippocampus

**Figure 2:**
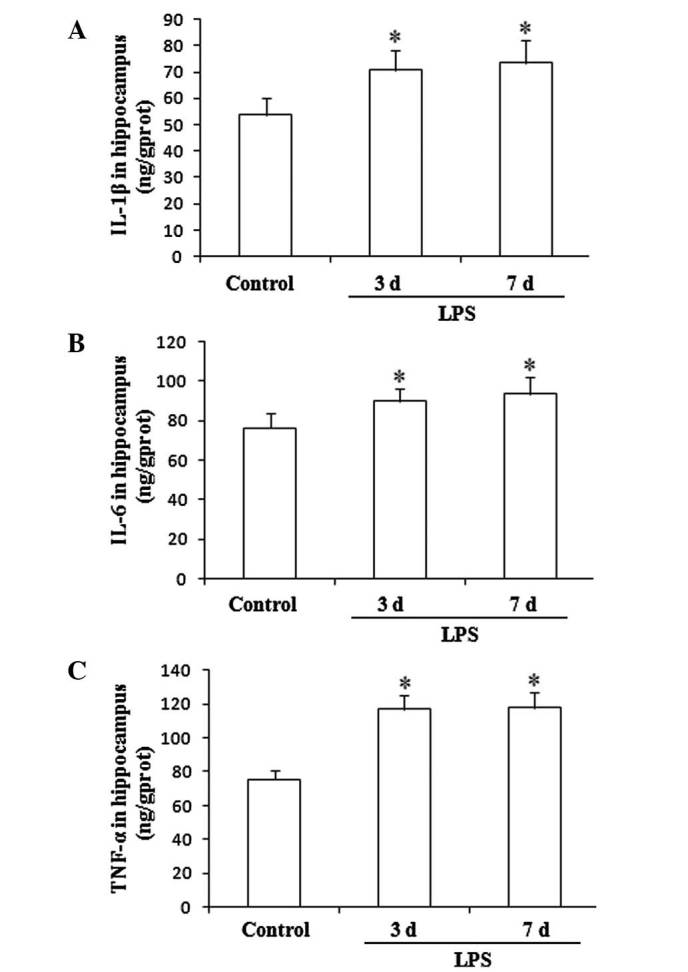
Effect of LPS on the expression levels of (A) IL-1β, (B) IL-6 and (C) TNF-α in the rat hippocampus. ^*^P<0.05, vs. control, ^#^P<0.05, vs. LPS 3 d. LPS, lipopolysaccharide; IL, interleukin; TNF, tumor necrosis factor.

The hippocampal expression levels of IL-1β, IL-6 and TNF-α showed significant increases in the rats undergoing LPS administration for 3 and 7 consecutive days, compared with the levels in the control group. (F_(2,27)_, 26.21, P<0.01; Fig. 2).

### Expression levels of Aβ in the hippocampus

**Figure 3:**
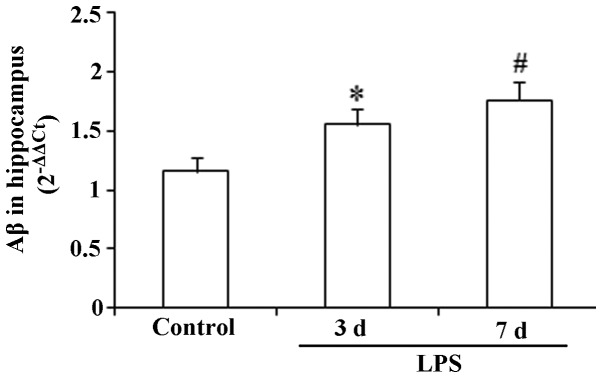
Effect of LPS on the expression level of Aβ in the rat hippocampus. ^*^P<0.05, vs. control. LPS, lipopolysaccharide; Aβ, amyloid-β.

As demonstrated in Fig. 3, no significant change in the hippocampal expression level of Aβ was observed following the administration of LPS for three consecutive days compared with that in the control group (P>0.05). However, 7 consecutive days of LPS administration induced a significant increase in the expression level of Aβ compared with that in the control (F_(2,27)_, 9.87; P<0.05).

### Expression levels of BDNF in the hippocampus

**Figure 4:**
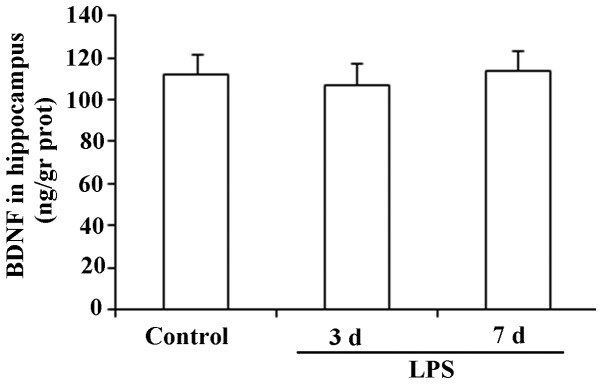
Effect of LPS on the expression level of BDNF in the rat hippocampus. LPS, lipopolysaccharide; BDNF, brain-derived neurotrophic factor.

The results presented in Fig. 4 show that there were no significant changes in the expression levels of BDNF in the hippocampus following the administration of LPS for 3 or 7 consecutive days (F_(2,27)_, 1.43; P>0.05).

## Discussion

The results of the Morris water test conducted in the present study demonstrate that intraperitoneally administered LPS elicited cognitive dysfunction in rats. Moreover, it was observed that LPS significantly increased the expression levels of pro-inflammatory cytokines and Aβ in the hippocampus. However, the previously expected reduction in the expression levels of BDNF was not observed.

In the Morris water maze, latency to the platform and the proportion of time spent in the target quadrant are two important testing indices for evaluating cognitive function in a rat model. In the present study, the results demonstrated that the chronic administration of LPS significantly increased the latency to the platform and decreased the proportion of time spent in the target quadrant, indicating that LPS elicited a deficit in cognitive performance.

LPS is a key component of the cell wall in Gram-negative bacteria and has the potential to cause sepsis, shock and microcirculation disturbance (20). Increasing evidence has shown that LPS may be administered to construct animal models of neurological diseases (21). Shaw *et al* (22) indicated that a single administration of LPS elicited cognitive impairments. In the present study, rats were intraperitoneally injected with LPS for 3 or 7 days in order to observe its effects on cognitive performance. Dantzer *et al* (9) hypothesized that following a single injection of LPS, particularly a short time after the administration, animals exhibit sickness behavior rather than cognitive impairment.

Pro-inflammatory cytokines are regulators of host responses to infection, immune responses, inflammation and trauma and worsen disease progression. A study by Leung *et al* (23) showed that increased levels of pro-inflammatory cytokines in the brain were associated with the pathogenesis of cognitive disturbance in patients with Alzheimer’s disease. Moreover, a previous study indicated that the increased expression of pro-inflammatory cytokines facilitated the emergence of cognitive impairments (24). These observations indicate that pro-inflammatory cytokines play a pivotal role in the pathogenesis of specific diseases characterized by cognitive impairments. In the present study, the expression levels of hippocampal IL-1β, IL-6 and TNF-α were observed and showed a significant increase following the chronic administration of LPS. Therefore, the results are consistent with previous observations.

Aβ is a component of the amyloid plaques that are associated with Alzheimer’s disease (25). Aβ is a highly multifunctional peptide with significant non-pathological activity (26). In the present study, increased expression of Aβ in the hippocampus was observed following the chronic administration of LPS, indicating that the increased expression of Aβ may be a major factor in the pathogenesis of cognitive dysfunction. Notably, administration of LPS for 7 days elicited an increase in the expression level of Aβ, while 3 days of LPS administration did not. The chronic administration of LPS increased the expression levels of pro-inflammatory cytokines in the hippocampus. Therefore, it was hypothesized that high expression levels of Aβ may be associated with increased pro-inflammatory cytokine levels in the hippocampus. In other words, long-term infection in the central nervous system may upregulate the expression of Aβ. Conversely, inhibiting the inflammatory response may facilitate the downregulation of Aβ (26). The results of the present study indicate that chronic LPS administration eliciting the upregulation of Aβ in rat hippocampus may be associated with the observed increase in the levels of pro-inflammatory cytokines.

BDNF is a member of the neurotrophin family of growth factors, which act on certain neurons of the central and peripheral nervous system (27). Lapchak *et al* (28) demonstrated that the administration of LPS reduced BDNF mRNA expression levels in the rat hippocampus. Moreover, several other studies have demonstrated that proinflammatory cytokines inhibit BDNF expression in the brain (29,30). Schnydrig *et al* (31) reported that synaptosomal BDNF expression levels in mice showed a transient reduction following the intraperitoneal administration of a single high dose of LPS, with a maximal reduction at day 3. Although previous studies have reported that BDNF plays a critical role in cognitive function, in the present study, changes in BDNF expression levels following LPS administration were not observed. Regarding the reason for this, it was considered that the LPS-elicited cognitive impairment animal model was not associated with changed BDNF expression levels. Due to this limitation, the possibility that intracranial injections of BDNF improve cognitive performance in LPS-induced cognitive impairment animal models was not investigated. Future studies are required to further investigate the correlation between LPS-induced cognitive impairment and BDNF expression.

In conclusion, LPS-induced cognitive dysfunction is likely to be associated with pro-inflammatory cytokines and Aβ. The results of the present study indirectly indicate that early intervention against the inflammatory responses may be a strategy for attenuating the increased expression of hippocampal Aβ. However, the present study did not investigate drug treatments that have the potential to intervene in the expression of pro-inflammatory cytokines and ultimately reverse the emergence of cognitive dysfunction. Consequently, future large-scale studies are required to further explain the pathogenesis of cognitive dysfunction.

## References

[1] Adam N, Kandelman S, Mantz J, Chrétien F, Sharshar T (2013). Sepsis-induced brain dysfunction. Expert Rev Anti Infect Ther.

[2] Zampieri FG, Park M, Machado FS, Azevedo LC (2011). Sepsis-associated encephalopathy: not just delirium. Clinics (Sao Paulo).

[3] Guan Z, Fang J (2006). Peripheral immune activation by lipopolysaccharide decreases neurotrophins in the cortex and hippocampus in rats. Brain Behav Immun.

[4] Suzumura A, Takeuchi H, Zhang G, Kuno R, Mizuno T (2006). Roles of glia-derived cytokines on neuronal degeneration and regeneration. Ann NY Acad Sci.

[5] Wang Y, Cui XL, Liu YF (2011). LPS inhibits the effects of fluoxetine on depression-like behavior and hippocampal neurogenesis in rats. Prog Neuropsychopharmacol Biol Psychiatry.

[6] Deng XH, Ai WM, Lei DL, Luo XG, Yan XX, Li Z (2012). Lipopolysaccharide induces paired immunoglobulin-like receptor B (PirB) expression, synaptic alteration, and learning-memory deficit in rats. Neuroscience.

[7] Richwine AF, Sparkman NL, Dilger RN, Buchanan JB, Johnson RW (2009). Cognitive deficits in interleukin-10-deficient mice after peripheral injection of lipopolysaccharide. Brain Behav Immun.

[8] Sparkman NL, Buchanan JB, Heyen JR, Chen J, Beverly JL, Johnson RW (2006). Interleukin-6 facilitates lipopolysaccharide-induced disruption in working memory and expression of other proinflammatory cytokines in hippocampal neuronal cell layers. J Neurosci.

[9] Dantzer R, O’Connor JC, Freund GG, Johnson RW, Kelley KW (2008). From inflammation to sickness and depression: when the immune system subjugates the brain. Nat Rev Neurosci.

[10] Smith CJ, Emsley HC, Udeh CT (2012). Interleukin-1 receptor antagonist reverses stroke-associated peripheral immune suppression. Cytokine.

[11] Krzyszton CP, Sparkman NL, Grant RW (2008). Exacerbated fatigue and motor deficits in interleukin-10-deficient mice after peripheral immune stimulation. Am J Physiol Regul Integr Comp Physiol.

[12] Johnston H, Boutin H, Allan SM (2011). Assessing the contribution of inflammation in models of Alzheimer’s disease. Biochem Soc Trans.

[13] Kaster MP, Gadotti VM, Calixto JB, Santos AR, Rodrigues AL (2012). Depressive-like behavior induced by tumor necrosis factor-α in mice. Neuropharmacology.

[14] Mansur RB, Zugman A, Asevedo EM, da Cunha GR, Bressan RA, Brietzke E (2012). Cytokines in schizophrenia: possible role of anti-inflammatory medications in clinical and preclinical stages. Psychiatry Clin Neurosci.

[15] Pizza V, Agresta A, D’Acunto CW, Festa M, Capasso A (2011). Neuroinflamm-aging and neurodegenerative diseases: an overview. CNS Neurol Disord Drug Targets.

[16] Magaki S, Mueller C, Dickson C, Kirsch W (2007). Increased production of inflammatory cytokines in mild cognitive impairment. Exp Gerontol.

[17] Oral E, Canpolat S, Yildirim S, Gulec M, Aliyev E, Aydin N (2012). Cognitive functions and serum levels of brain-derived neurotrophic factor in patients with major depressive disorder. Brain Res Bull.

[18] Zhang XY, Liang J, Chen da C (2012). Low BDNF is associated with cognitive impairment in chronic patients with schizophrenia. Psychopharmacology (Berl).

[19] D’Hooge R, De Deyn PP (2001). Applications of the Morris water maze in the study of learning and memory. Brain Res Brain Res Rev.

[20] Solov’eva T, Davydova V, Krasikova I, Yermak I (2013). Marine compounds with therapeutic potential in gram-negative sepsis. Mar Drugs.

[21] Skelly DT, Hennessy E, Dansereau MA, Cunningham C (2013). A systematic analysis of the peripheral and CNS effects of systemic LPS, IL-1β, TNF-α and IL-6 challenges in C57BL/6 mice. PLoS One.

[22] Shaw KN, Commins S, O’Mara SM (2001). Lipopolysaccharide causes deficits in spatial learning in the watermaze but not in BDNF expression in the rat dentate gyrus. Behav Brain Res.

[23] Leung R, Proitsi P, Simmons A (2013). Inflammatory proteins in plasma are associated with severity of Alzheimer’s disease. PLoS One.

[24] Reale M, Iarlori C, Gambi F (2004). Treatment with an acetylcholinesterase inhibitor in Alzheimer patients modulates the expression and production of the pro-inflammatory and anti-inflammatory cytokines. J Neuroimmunol.

[25] Hsu LJ, Mallory M, Xia Y (1998). Expression pattern of synucleins (non-Abeta component of Alzheimer’s disease amyloid precursor protein/alpha-synuclein) during murine brain development. J Neurochem.

[26] Nguyen JT, Yamani A, Kiso Y (2006). Views on amyloid hypothesis and secretase inhibitors for treating Alzheimer’s disease: progress and problems. Curr Pharm Des.

[27] Yan Q, Rosenfeld RD, Matheson CR (1997). Expression of brain-derived neurotrophic factor protein in the adult rat central nervous system. Neuroscience.

[28] Lapchak PA, Araujo DM, Hefti F (1993). Systemic interleukin-1 beta decreases brain-derived neurotrophic factor messenger RNA expression in the rat hippocampal formation. Neuroscience.

[29] Maher FO, Martin DS, Lynch MA (2004). Increased IL-1beta in cortex of aged rats is accompanied by downregulation of ERK and PI-3 kinase. Neurobiol Aging.

[30] Taishi P, Churchill L, De A, Obal F, Krueger JM (2008). Cytokine mRNA induction by interleukin-1beta or tumor necrosis factor alpha in vitro and in vivo. Brain Res.

[31] Schnydrig S, Korner L, Landweer S (2007). Peripheral lipopolysaccharide administration transiently affects expression of brain-derived neurotrophic factor, corticotropin and proopiomelanocortin in mouse brain. Neurosci Lett.

